# Reparative and Regenerative Effects of Mesenchymal Stromal Cells—Promising Potential for Kidney Transplantation?

**DOI:** 10.3390/ijms20184614

**Published:** 2019-09-18

**Authors:** Merel Pool, Henri Leuvenink, Cyril Moers

**Affiliations:** Department of Surgery–Organ Donation and Transplantation, University Medical Center Groningen, 9713GZ Groningen, The Netherlands; h.g.d.leuvenink@umcg.nl (H.L.); c.moers@umcg.nl (C.M.)

**Keywords:** mesenchymal stromal cells, regeneration, kidney transplantation

## Abstract

Mesenchymal stromal cells (MSCs) possess reparative, regenerative and immunomodulatory properties. The current literature suggests that MSCs could improve kidney transplant outcome via immunomodulation. In many clinical domains, research has also focussed on the regenerative and reparative effects of therapies with MSCs. However, in the field of transplantation, data on this subject remain scarce. This review provides an overview of what is known about the regenerative and reparative effects of MSCs in various fields ranging from wound care to fracture healing and also examines the potential of these promising MSC properties to improve the outcome of kidney transplantations.

## 1. Introduction

Extensive research has been conducted on the unique immunomodulatory and regenerative properties of mesenchymal stromal cells (MSCs) [1]. The promising combination of tissue regeneration and immune modulation represents a great potential for the field of organ transplantation. Renal transplantation is limited by the shortage of donor kidneys [2]. In an attempt to decrease waiting time by enlargement of the deceased organ donor pool, an increasing number of organs from donation after circulatory death (DCD) and from extended criteria donors (ECD) are being used [3]. A drawback of such donor kidneys is that they are of inferior quality and are more prone to ischaemia–reperfusion injury. MSCs could play an important role in pre-transplant ex-vivo reconditioning, post-transplant in vivo damage repair and immunomodulation, allowing to increase the long-term survival of these DCD and ECD grafts. So far, most research effort has been focussed on immunomodulatory effects of MSCs after transplantation. Data on the regenerative properties of MSCs, which may help to repair damaged donor organs, are still scarce. The aim of this review is to give an overview of what is known about the reparative and regenerative effects of MSCs in order to evaluate how these properties could be advantageous in the field of kidney transplantation.

## 2. Characteristics of Mesenchymal Stromal Cells (MSCs)

MSCs are multipotent cells that can be isolated from different sources [4]. Their distinctiveness relies on the following criteria: adherence to plastic; the potential to differentiate into adipocytes, chondrocytes and osteoblasts; the expression of markers such as CD73, CD90 and CD105 and the lack of expression of markers CD31, CD34 and CD45 [5]. MSCs can be expanded in vitro whilst retaining a relatively stable phenotype, thus creating the opportunity to culture large numbers of cells for clinical use [6]. MSCs have the ability to home to areas of injury or inflammation and modulate innate as well as adaptive immune responses [5,7]. Furthermore, MSCs are reported to play an important role in tissue repair and regeneration. Most likely, they achieve this via paracrine and endocrine signals that exert anti-inflammatory, anti-apoptotic and pro-angiogenic actions [5].

## 3. Sources of MSCs

Major sources of MSCs are the bone marrow (BM-MSC), the adipose tissue (A-MSC) and peripheral blood. MSCs can also be isolated from umbilical cord (UC-MSC), umbilical cord blood (CB-MSC), urine, amnion and placenta [8,9]. The number of MSCs that can be acquired per isolation varies depending on the source [10]. BM-MSCs were the first to be discovered and therefore are the most studied type of MSCs. They often serve as the gold standard and were initially used in most clinical trials [11]. Nowadays, an increasing number of clinical trials use A-MSCs or UC-MSCs. Genetic differences and variations in cell surface marker expression between MSCs from different sources, especially between A-MSCs and BM-MSCs, have been thoroughly studied as have the differences between cytokine and chemokine production of these cells [11,12,13,14].

As pigs have genetic traits similar to those of humans, the use of porcine MSCs (pMSCs) for treatment in post-mortem kidney donation might be an interesting clinical option in the future. The fact that MSCs are poorly recognisable by the immune system potentially renders them safe and suitable for xenotransplantation purposes. The therapeutic potential of pMSCs has been studied in various animal models and it has been shown that these cells function across the xenogeneic barrier without adverse reactions occurring [15,16].

As most research has been conducted using only one source of MSCs, very few analyses are available which directly compare the efficacy of different types of MSCs. The scarce studies that have been performed comparing A-MSCs and BM-MSCs showed that A-MSCs might have a stronger immunosuppressive potential than BM-MSCs [17,18]. Although this might be the case in vitro, further research is necessary to see if these cells behave in the same manner in vivo. Furthermore, these studies did not specifically report on the regenerative properties of different types of MSCs. An advantage of A-MSCs is that the harvesting approach is considered to be safe, and a significantly higher number of cells can be obtained from the same amount of tissue, compared to BM-MSCs [19]. These advantages also apply to UC-MSCs, as they are harvested from tissue which is otherwise discarded at birth [20].

## 4. Environmental Effects on MSCs

MSCs can be activated into two different phenotypes: a pro-inflammatory or an immuno-suppressive phenotype [21]. The environment in which MSCs interact with T cells determines the effect MSCs have on T cells. In a pro-inflammatory environment, with high concentrations of interferon-γ (IFN-γ), interleukin-2 or tumour necrosis factor-α (TNF-α), inhibition of T cell proliferation occurs, whereas in anti-inflammatory environments this does not happen [22]. When T cells are cultured together with MSCs, T regulatory cells (Tregs) increase [23].

There is also evidence that inflammatory stimuIi can induce the release of exosomes by MSCs. These exosomes are believed to lead to similar reparative and regenerative effects on tissues as those of MSCs [24]. These findings are in line with unpublished results from our group in which we found an upregulation of pro-inflammatory cytokines when ischaemically damaged porcine kidneys were treated with MSCs. Therefore, it cannot be concluded that the immunosuppressive phenotype of MSC is the property that plays a central role in tissue repair and regeneration.

## 5. Allogeneic Versus Autologous MSCs

MSCs could have a great therapeutic potential in the field of organ transplantation. In the case of an organ transplantation, allogeneicity is inevitable. The question arises whether administration of allogeneic MSCs is preferable to that of autologous MSCs. Treatment with allogeneic MSCs offers the advantage of a more standard consistency of the product, but there are always concerns regarding the body’s response to allogeneic products [25]. Logistically, the usage of autologous MSCs seems challenging, but in the case of living kidney donation, these cells could be harvested from each donor, cultured and stored in liquid nitrogen until they are needed. As the median age of patients on the waiting list for a kidney transplant is rising and the average number of tissue-resident A-MSCs, in contrast to that of BM-MSCs, does not decline with rising age, the use of A-MSCs might be preferable when considering treatment with autologous MSCs in a transplant setting [2,10,26,27].

In post-mortem kidney donation, the acute setting does not allow for these procedures, thus allogeneic MSCs would have to be used. Because of their immunosuppressive properties and low immunogenicity in comparison with other cell types, allogeneic-MSCs are often used for cell therapies. Despite two studies reporting immune responses leading to, respectively, faster skin allograft and kidney rejection in rats treated with allogeneic MSCs in comparison with the control group that did not receive MSCs, treatment with allogeneic MSCs still remains a promising option, as most studies report a beneficial effect [28,29,30]. A few studies have directly compared the effects of autologous and allogeneic MSCs. One study, in which acute kidney injury was treated with MSCs, showed that identical doses of allogeneic MSCs were less effective than autologous MSCs [31]. On the contrary, in other fields, such as that of the treatment of non-ischaemic dilated cardiomyopathy as well as acute myocardial infarction, there is evidence that allogeneic BM-MSCs are even more effective than autologous BM-MSCs [32]. However, an important side note is that most patients suffering from these conditions are of older age. As we know that the number and function of the BM-MSCs decline with age, this could also explain the difference seen between autologous and allogeneic BM-MSCs [33]. In orthopaedics, specifically in prophylactic and curative treatment of arthritis, the beneficial effect of allogeneic and xenogeneic MSCs has also been proven [34]. Further research is necessary to conclude whether autologous or allogeneic MSCs are most suitable for a transplant setting.

## 6. MSC Therapy in Non-Transplant-Related Fields

### 6.1. Wound Healing and Angiogenesis

Wound healing is a complex process involving many steps such as wound closure, inflammation, re-epithelialisation and angiogenesis. The beneficial effect that MSCs have on this process seem to be mediated via paracrine interactions [35]. Several studies have demonstrated that MSCs accelerate wound closure by increasing the migration of fibroblasts [36,37]. Angiogenesis, vasculogenesis and arteriogenesis are three mechanisms which lead to blood vessel regeneration. By releasing proteases and angiogenic factors, MSCs have been reported to stimulate these mechanisms [38].

Liu et al. showed in a rat model that the treatment of burn wounds with UC-MSCs decreased the wound healing time and was associated with higher levels of vascular endothelial growth factor (VEGF), a higher number of microvessels and an elevated cutaneous wound microcirculation [39]. In a mouse model, Luo et al. found that the administration of umbilical cord blood MSCs to mice with a full skin defect led to a thicker newly formed epidermis layer, increased dermal ridges, increased the number of cells in the regenerated skin tissue and promoted a more regular alignment of fibres in the skin tissue in comparison with the control group [40]. Matrix metalloprotease-1 (MMP-1) and fibronectin affect collagen components in the dermis. Jeon et al. showed that in rats with skin defects, the injection of conditioned media derived from culturing human CB-MSCs was associated with lower protein expression and lower total levels of MMP-1, as well as higher expression of fibronectin in comparison with the controls. As MMP-1 is known to play a prominent role in the modification and degradation of the extracellular matrix, this finding strongly suggests that MSC preserve the collagenous matrix [37]. In mice with a skin defect to the fascial level, intravenous infusion of human BM-MSCs in combination with a locally administered polymer containing BM-MSCs, led to healing of the skin without retraction and scar formation. In comparison with the control group, less scar formation was also seen in mice only receiving intravenous infusion of BM-MSCs [41]. There is evidence that BM-MSCs also enhance wound healing in diabetic mice [42]. Administration of such MSCs is considered as a novel approach towards assisted healing of chronic wounds, since current treatment options are largely ineffective.

### 6.2. Fracture Healing and Orthopaedics

Apart from the beneficial effect that MSCs can exert via paracrine interactions, they are also known to be able to differentiate into multiple cell lineages. However, they do not possess the plasticity that is typical of embryonic stem cells. The exact differentiation process of MSCs is not completely clear [43]. Multiple factors play a role in stimulating MSCs to form bone and cartilage precursors. Apart from the mechanical environment, bone morphogenetic protein 4 and bone morphogenetic protein 2 have been shown to be strong stimulators of this differentiation [44,45].

Fracture healing is unique, as complete regeneration often occurs, and the newly formed bone is indistinguishable from the uninjured bone [43]. However, in some cases, complications arise such as a hypertrophic or atrophic non-unions. Hernigou et al. injected bone marrow into atrophic non-unions of the tibia. and bone union was reached in most patients. However, the efficacy of the percutaneous bone marrow grafting was dependent on the number of progenitor cells in the graft and in the harvested bone marrow aspirate [46]. Granero-Moltó and colleagues used a tibia fracture mouse model to show that MSCs enhanced fracture healing. Transplanted MSCs migrated to the fracture site and improved the biomechanical properties of the callus. Size, cartilaginous and bone content of the fracture callus were increased [47].

The application of MSCs for osteonecrosis of the femoral head (OFNH) has also been investigated. OFNH is a condition in which the death of osteocytes leads to structural changes, subsequently leading to the collapse of the femoral head. Some contradictory findings have been published about the effect of BM-MSC treatment on different stages of ONFH. Multiple studies showed that BM-MSC decreased the volume of necrotic lesions in patients with early-stage ONFH and led to a significant improvement of the symptoms. However, there is evidence that treatment in later stages with BM-MSCs leads to failing clinical results. Therefore, the general conclusion is that BM-MSC treatment seems to have a beneficial effect mainly in early stages of ONFH, regarding symptomatic relief and prevention of the progression of femoral head collapse [48].

### 6.3. Neuronal Differentiation

The earliest studies obtained insight in the ability of MSCs to differentiate into adipocytes and bone tissue. Further studies have shed light on their differentiation into myocytes, tenocytes and neurons, under specific in vitro conditions [49]. The first protocols on neuronal differentiation used toxic drugs to stimulate MSCs to acquire a neuronal-like morphology and elicit specific neuronal cell surface marker expression. However, subsequent studies showed that these changes were most likely due to stress rather than actual differentiation [50].

Takeda and Xu treated MSCs with exosomes derived from a neuronal cell line. These MSCs acquired a neuronal-like morphology, and protein and gene expression of several neuronal markers was upregulated [51]. Using another protocol, Woodbury et al. also showed that stromal cells expressed neuron-specific markers after exosome treatment [52]. However, in order to be identified and function as a neuron, an MSC-derived cell must show synaptic transmission, have a resting membrane potential and be able to generate action potentials [50]. Kohyama et al. succeeded in generating functional mature neurons that responded to depolarising stimuli [53]. On the contrary, Hofstetter and colleagues demonstrated that although their neuron-like cells exhibited certain neuronal morphologies, they lacked voltage-gated ion channels, thus being unable to generate action potentials. In addition, this group discovered that the implantation of MSCs into an injured spinal cord improved recovery by forming bundling bridges across the lesion [54].

### 6.4. Atherosclerotic Renal Vascular Disease

Renal blood flow is reduced in atherosclerotic renovascular disease. Eventually, this impairment in blood flow will result in vascular rarefaction, oxidative stress and inflammation [55]. Preclinical research has shown that in pigs with atherosclerotic renal artery stenosis, the combination of renal revascularisation and infusion of MSCs resulted in restored renal function, increased microvascular density and decreased inflammation and fibrosis [56,57]. Saad et al. investigated the efficacy and safety of intra-arterial infusion of A-MSCs in humans suffering from renal vascular disease. They concluded that the infusion did not produce any adverse effects and led to increased cortical perfusion of the kidney, probably as a result of proangiogenic factors released by the MSCs [55].

### 6.5. Acute Kidney Injury

Unlike many other organs, kidneys are capable of cell proliferation and repair after ischaemic or toxic injury. Normally, cell turnover in the kidney is extremely low, but when tubules are injured, a sudden increase in cell proliferation of the surviving tubular epithelial cells can be seen, which in turn leads to the restoration of tubular integrity by replacing the injured cells [58]. Acute kidney injury (AKI) is a disease with a mixed aetiology, which is defined by an acute decrease in kidney function as a result of structural injury [59]. The interest in MSCs as a cell therapy treatment in AKI started to grow once their cellular plasticity became evident. Although an initial study reported that BM-MSCs transdifferentiated into renal epithelium, these findings could not be repeated in follow-up studies [60]. Recent studies have shown that instead of transdifferentiation and directly replacement of dead tubular cells, there is a certain degree of cell fusion between MSCs and the tubular epithelium. Furthermore, in most studies the protective and reparative effect of MSCs was observed in 1 to 2 days, which seems too rapid to be explained by MSCs differentiating into renal tubular epithelium [61]. In a mouse model, intravenous infusion of human BM-MSCs reduced epithelial cell injury of the proximal tubules and cell apoptosis, increased renal cell proliferation and prolonged cell survival in cisplatin-induced AKI [62]. There is also evidence that the beneficial effect of MSCs in AKI relies on inflammatory response modulation. Semedo et al. showed that in their model of AKI in rats, animals treated with MSCs had lowest levels of serum creatinine and faster tissue regeneration in comparison with untreated rats. The immunomodulatory effects exerted by MSCs were measured after 24 h, revealing a higher expression of Th2 cytokines (interleukin-10 (IL-10) and interleukin-4) as well as a lower expression of Th1 cytokines (TNF-α, interleukin-6 (IL-6) and interleukin-1 beta). After 48 h, this balance had already shifted, therefore it can be concluded that early modulation of the inflammatory response has a significant beneficial effect on the injured kidney [63].

Oxidative metabolism by mitochondria is a process on which the kidney relies to provide the necessary adenosine triphosphate for tubular reabsorption. Therefore, mitochondrial dysfunction seen in AKI plays a central role in the pathophysiological changes, either as a contributor or as an initiator. There is evidence that, via endocrine and paracrine mechanisms, MSCs are able to protect renal cells from mitochondrion-related apoptosis and stimulate the recovery of function, mass and density of the mitochondria and could therefore play an important role in acute kidney injury management [64,65].

## 7. MSCs in Kidney Transplantation

To date, only a few preclinical studies have been performed using MSCs in combination with renal transplantation. Yu et al. performed kidney transplants in rats and injected these rats postoperatively with BM-MSCs. The renal grafts of these rats showed less interstitial fibrosis and inflammation, as well as reduction of glomerulosclerosis in comparison with rats in the control group [66]. A study by Gregorini et al., in which MSCs/MSC-derived extracellular vesicles were added during ex-vivo hypothermic machine perfusion of isolated rat kidneys, showed that overall these kidneys had less ischaemic damage. The levels of pyruvate were higher, and those of lactate dehydrogenase (LDH), malondialdehyde (MDA) and lactate were lower in the kidneys that received this therapy. In addition, several genes associated with the improvement of cellular energy metabolism were upregulated, as well as several genes encoding for proteins which play a role in the membrane transport of ions [67]. In another study, Gregorini et al. demonstrated that the injection of MSCs into the renal artery directly after reperfusion of the transplanted kidney, led to a rise in the levels of IL-10 and a decrease in the serum levels of IL-6 and IFN-γ in comparison with rats which did not receive an MSC injection in the renal artery [68]. In a porcine model, the injection of autologous amniotic fluid-derived MSCs in the renal artery 6 days after transplantation led to improved function of glomeruli and tubules, as well as significantly less fibrosis after 3 months [69]. These results are in line with those of a study in which rats underwent a kidney transplantation and were injected with MSCs 11 weeks postoperatively. These renal grafts also showed less fibrosis and less atrophy of the tubules [70].

Over the past years, research into the therapeutic applications of MSCs has expanded drastically leading to the registration of hundreds of clinical trials. However, only 15 of these studies focus on kidney transplantation. The status of five of these studies has not been updated over the past 4 years, with their latest status being ‘not yet recruiting’. The 10 remaining clinical trials can be found in Table 1. These studies focus on the safety of treatment with MSCs as well as on the reduction of immunosuppressive medication but do not look into regenerative or reparative effects.

One of the few published studies in humans performed by Erpicum et al. in which patients received a single infusion of MSCs post-transplant led to improved estimated glomerular filtration rate (eGFR) in the first year after transplantation as well as to an increased number of Tregs [71]. Another study by Reinders et al. focussed on the influence of intravenously infused MSCs on allograft rejection. It showed that two infusions, 6 months after transplantation, of 1–2 million BM-MSCs per kilogram of body weight led to signs of systemic immunosuppression in kidney transplant recipients but it did not report on the potential regenerative effects of systemic MSC administration [72]. No studies have focussed on administering MSCs to the kidney ex-vivo, prior to transplantation. However, a study performed by Pacienza et al. in which MSCs were administered to donor lungs during warm ischaemia prior to normothermic lung perfusion, showed that MSCs protected the lungs from oxidative stress during ischaemia [73].

A lethal complication of solid-organ transplantation is graft-versus-host disease (GVHD). GVHD is the result of an immunologic reaction of donor T cells against host cells. It is known to be a major complication of allogeneic hematopoietic stem cell transplantation and has also been described in liver and small bowel transplant recipients. Even though it is a relatively uncommon complication after kidney transplantation, a few case reports can be found [74]. In 2004, Le Blanc et al. were the first to describe the successful treatment with MSCs in a child with steroid-resistant GVHD of liver and gut after allogeneic stem cell transplantation. The MSC treatment had an immunosuppressive effect and also a rapid reparative effect on the damaged gut epithelium [75]. In another study performed by this group, patients with tissue toxicity as a result of allogeneic hematopoietic stem cell transplantation received MSC treatment. This led to dramatic resolution of pneumomediastinum and haemorrhagic cystitis as well as of peritonitis caused by colonic perforation [76]. Resolution of intestinal perforation in patients with acute GVHD after treatment with MSCs has also been seen in other studies [77]. There is also evidence that MSC treatment could protect from GVHD development [78]. Organ transplant recipients who are in need of immune modulation or tissue repair or suffer from GVHD could benefit from the effects.

From the studies mentioned above, the potential beneficial effects of MSCs for kidney transplantation are listed in Table 2.

## 8. Administration, Survival, Preconditioning and Safety of MSCs

Liu et al. found that the timing of administration of MSCs is of vital importance for their potency. In vitro, when MSCs were administered within one hour after ischaemia–reperfusion injury, the highest protective and anti-inflammatory effects were observed. This finding suggests that MSCs should be administered prior to the peak of inflammation [79]. These results are in line with those of a study by Erpicum et al. in which rats were injected with MSCs 7 days prior to ischaemia/reperfusion or 1 day after ischaemia–reperfusion. In comparison with the control group and the group which received MSC treatment 1 day after ischaemia–reperfusion, the group which received MSCs prior to injury showed lower levels of pro-inflammatory cytokines, less cell apoptosis and less signs of tubular damage [80].

MSCs can be administered via different routes and, depending on this route, will localise to different, perhaps non-target, organs. In the case of an intravenous infusion, MSCs will circulate systemically, but the first capillaries encountered will be those of the pulmonary vascular bed. This is in contrast with administering them directly via the renal artery, which allows to reach directly the microcirculation of a certain target organ. The number of eventually surviving cells as well as their localisation are important factors for their protective and regenerative effects [81]. In order to increase the number of surviving cells and their efficacy, different pre-conditioning strategies are being investigated. A study by Putra et al. showed that in an AKI model in rats, hypoxia-preconditioned MSCs were more effective in improving the renal function than MSCs that were cultured under normal oxygen conditions [82]. In vitro studies in which hypoxia improved the proliferative abilities and survival of MSCs support these results [83]. Some research has also focussed on increasing the migratory ability of MSCs. Chemokine (C-X-C motif) receptor 4 (CXCR4) is known to play an important role in homing of stem cells and is expressed abundantly on hematopoietic stem cells but not on BM-MSCs. When MSCs are cultured together with certain chemical compounds, they express higher levels of CXCR4 [84]. A study by Xinaris et al. showed that this method increased the survival of MSCs and enhanced their migration to the site of injury, which resulted in structural repair in this AKI mouse model [85]. There is also evidence that MSCs preconditioned to the innate immune system by culturing them with cytokines present increased migration to the site of inflammation and are protected from natural killer (NK)-mediated cytotoxicity [86]. Another study in which MSCs were pre-treated with melatonin before administration to an AKI model in rats, also showed increased survival of MSCs, increased cell proliferation and angiogenesis, as well as quicker recovery of the renal function [87]. A neoadjuvant approach to stimulate homing of MSCs to areas of inflammation in AKI is the use of pulsed focused ultrasound (pFUS). A study by Burks et al. found that targeting the kidney with pFUS led to increased homing of MSCs and this in turn decreased cell death and improved the renal function compared to the treatment with MSCs alone [88]. These studies have shown that preconditioning of MSCs should be considered when using this therapy in kidney transplantation, as it has a significant beneficial effect on the survival and reparative and regenerative properties of these cells.

Initially, there were safety concerns regarding therapies with MSCs. As MSCs suppress the immune system and secrete many growth factors, they theoretically have the potential to enhance tumour formation and progression and increase susceptibility to infection. However, on the basis of current clinical trials with long-term follow-up, there is no association between treatment with MSCs and the occurrence of infections, the development of malignancies, organ system complications or death. However, a significant association was seen between administration of MSCs and transient fever, but the exact mechanism of this phenomenon has not been elucidated. As fever was not associated with adverse outcomes in these patients, therapy with MSCs in considered to be safe [89,90].

## 9. Conclusions

Numerous studies on the beneficial and regenerative effects of MSC treatment have been performed in various clinical fields. However, in kidney transplantation, a procedure which goes hand in hand with immunosuppression, most research has focussed solely on the immunomodulatory potential of MSCs. As we are accepting more and more donor kidneys of inferior quality, the focus of research in kidney transplantation is slowly shifting towards enhancing organ preservation, facilitating tissue repair and inducing graft regeneration. From studies in other fields, we can conclude that MSCs could play a significant role in the outcome of marginal-quality kidney transplantations by releasing factors decreasing fibrosis, reducing inflammation, improving the function of tubuli and glomeruli and increasing angiogenesis. As more transplant centres are experimenting with conditioning isolated donor kidneys ex-vivo, this pre-transplant time window could be the ideal moment to administer MSCs, as it precedes the peak of inflammation. In contrast to administering the MSCs to a recipient systemically, no host immune response is present during ex-vivo perfusion. Furthermore, since ex-vivo cell therapy is not administered to a whole body, lower doses of MSCs could suffice. Based on current literature, it seems preferable to use preconditioned MSCs in order to increase the number of surviving cells, their migratory capacity and their efficacy. Data on the difference in effect of allogeneic and autologous MSCs are scarce, and the studies that are available show contradictory results. Therefore, pre-clinical studies are necessary to determine if allogeneic MSCs are preferable to autologous MSCs in a transplant setting and whether these cells should be bone-marrow-or adipose tissue-derived.

## Figures and Tables

**Table 1:** The 10 registered clinical trials on the subject of mesenchymal stem/stromal cell therapy in kidney transplantation (clinicaltrials.gov–accessed 12 September 2019). MSCs: mesenchymal stromal cells, BM-MSC: bone marrow MSC, UC-MSC: umbilical cord MSC, DGF: delayed graft failure, RATG: rabbit-antithymocyte-globulin, SVF: stromal vascular fraction.

Status	Title	Location	Source	Outcome
terminated	Mesenchymal Stem Cells Under Basiliximab/Low-Dose RATG to Induce Renal Transplant Tolerance	Bergamo, Italy	Autologous BM-MSC	Safety of treatment;Percentage of inhibition of memory T cell response
completed	Allogeneic Mesenchymal Stromal Cell Therapy in Renal Transplant Recipients	Leiden, Netherlands	AllogeneicBM-MSC	Safety of treatment
completed	Induction Therapy With Autologous Mesenchymal Stem Cells for Kidney Allografts	Fuzhou, China	Autologous BM-MSC	Evaluate MSCs as an alternative for antibody induction therapy
unknown	Induction With SVF-Derived MSC in Living-Related Kidney Transplantation	Fuzhou, China	Autologous stromal vascular fraction	Effective reduction of post-transplant immunosuppressive drugs
unknown	A Perspective Multicentre Controlled Study On the Application Of Mesenchymal Stem Cell To Prevent Rejection After Renal Transplantation By Donation After Cardiac Death	Guangzhou, China	Allogeneic UC-MSC	Reduction of rejection and DGF after renal transplantation
recruiting	Mesenchymal Stromal Cell Therapy in Renal Recipients	Leiden, Netherlands	Autologous BM-MSC	Reduction of fibrosis as well as facilitation of tacrolimus withdrawal
recruiting	Tolerance by Engaging Antigen During Cellular Homeostasis	North Carolina, United States	Autologous BM-MSC	Safety of treatment;Reduction of immunosuppressive drugs
recruiting	Mesenchymal Stromal Cells in Kidney Transplant Recipients	Bergamo, Italy	Autologous BM-MSC	Induce tolerance in living donor recipients
recruiting	MSC and Kidney Transplant Tolerance (Phase A)	Bergamo, Italy	Allogeneic BM-MSC	Induce tolerance in recipients of deceased donor kidneys
recruiting	Mesenchymal Stromal Cells in Living-Donor Kidney Transplantation	Houston, United States	Autologous source not specified	Safety of treatment;Reduction of immunosuppressive drugs

**Table 2:** Eight potential beneficial effects of MSCs in kidney transplantation.

Eight Potential Beneficial Effects of MSCs in Kidney Transplantation			
I.	Angiogenesis/arteriogenesis [25,38]	V.	Increase in T regulatory cells [16]
II.	Decreased fibrosis [39,52,53]	VI.	Inhibition of cytotoxic T cell proliferation [15]
III.	Decreased inflammation [39,40,51]	VII.	Less tissue damage (lower LDA and MDA) [50]
IV.	Improved function of glomeruli and tubuli [52]	VIII.	Upregulation of genes encoding proteins involved in improved membrane transport of ions [50]

## Abbreviations

AKI	acute kidney injury
A-MSC	adipose tissue mesenchymal stromal cells
BM-MSC	bone-marrow mesenchymal stromal cells
CB-MSC	cord blood mesenchymal stromal cells
CXCR4	chemokine (C-X-C motif) receptor 4
DCD	donation after cardiac death
DGF	delayed graft function
ECD	extended criteria donors
IFN-γ	interferon gamma
IL-6	interleukin-6
IL-10	interleukin-10
LDH	lactate dehydrogenase
MDA	malondialdehyde
MMP-1	matrix metalloprotease-1
MSC	mesenchymal stromal cell
OFNH	osteonecrosis of the femoral head
pFUS	pulsed focused ultrasound
pMSC	porcine mesenchymal stromal cells
RATG	rabbit-antithymocyte-globulin
SVF	stromal vascular fraction
TNF-α	tumour necrosis factor alpha
Treg	T regulatory cell
UC-MSC	umbilical cord mesenchymal stromal cells
